# Supplementary material for: Multicolor recordable and erasable photonic crystals based on on-off thermoswitchable mechanochromism toward inkless rewritable paper
Source: Nat Commun. 2024 Jul 5;15:5643. doi: 10.1038/s41467-024-49860-3 (PMC11226673; doi:10.1038/s41467-024-49860-3)
Supplement: Supplementary file 1 — Supplementary Information [file 41467_2024_49860_MOESM1_ESM.pdf]

Supplementary Information

Multicolor recordable and erasable photonic crystals based on on-off thermoswitchable mechanochromism toward inkless rewritable paper

Yang Hu[1], Chenze Qi[1], Dekun Ma[1], Dongpeng Yang[1]*, and Shaoming Huang[2]*

[1] Zhejiang Key Laboratory of Alternative Technologies for Fine Chemicals Process, School of Chemistry and Chemical Engineering, Shaoxing University, Shaoxing 312000, China

[2] School of Chemistry and Materials Science, Hangzhou Institute for Advanced Study, University of Chinese Academy of Sciences, Hangzhou, 310024, China.

E-mail: dpyang@usx.edu.cn (D. P. Yang); smhuang@gdut.edu.cn (S. M. Huang)

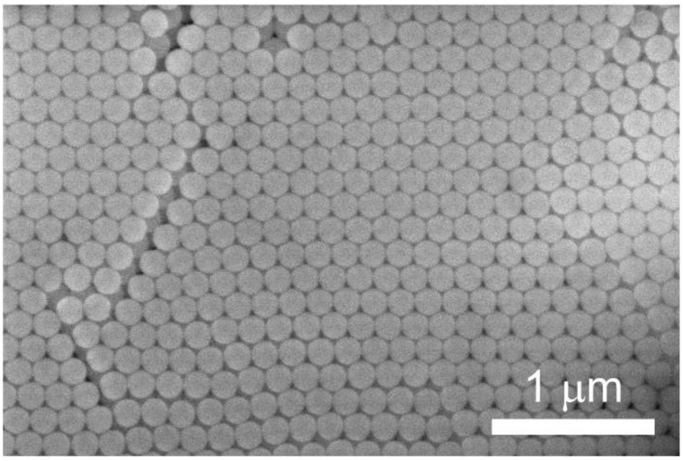

Supplementary Figure 1. Characterization of silica particles. SEM image of silica particles with a diameter of 167 nm.

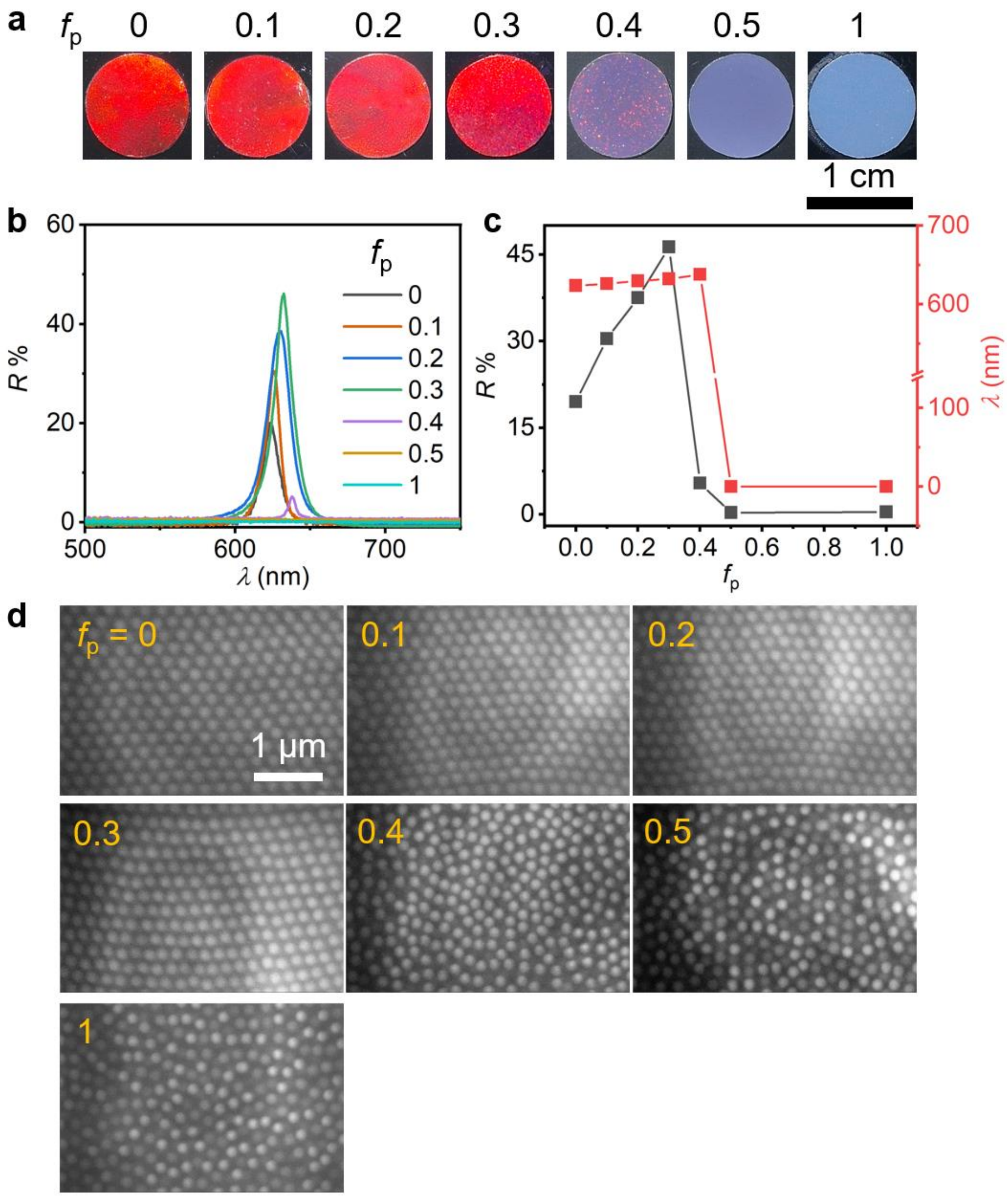

Supplementary Figure 2. The effect of f_p on MPCs' reflectance. a Digital photos and **b** corresponding reflection spectra of MPCs with different f_p. **c** Reflectance and wavelength of the MPC as the function of f_p. **d** The SEM images of the MPC with diverse f_p.

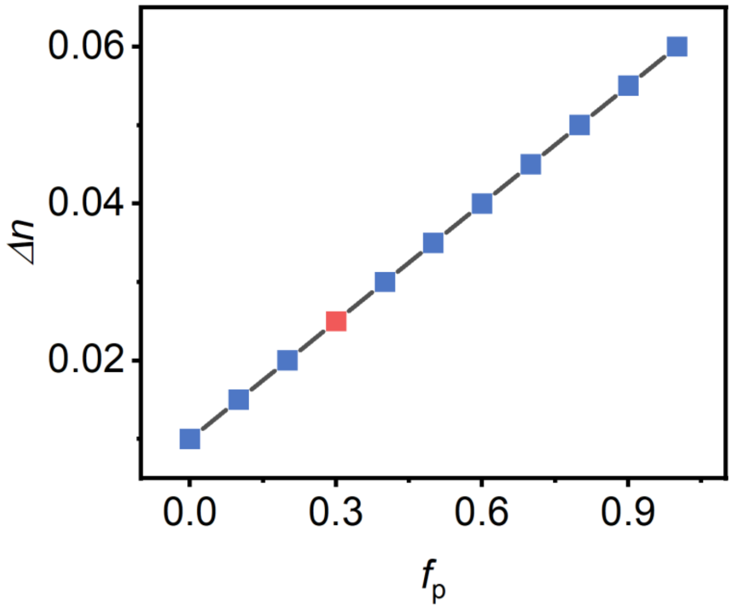

Supplementary Figure 3. *Δn* analysis. The *Δn* as a function of f_p.

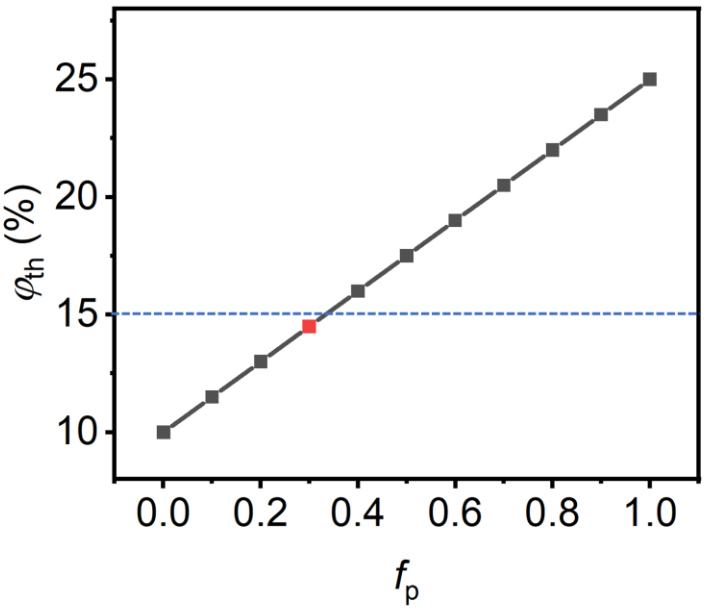

Supplementary Figure 4. φ_{th} analysis. The φ_{th} as a function of f_p.

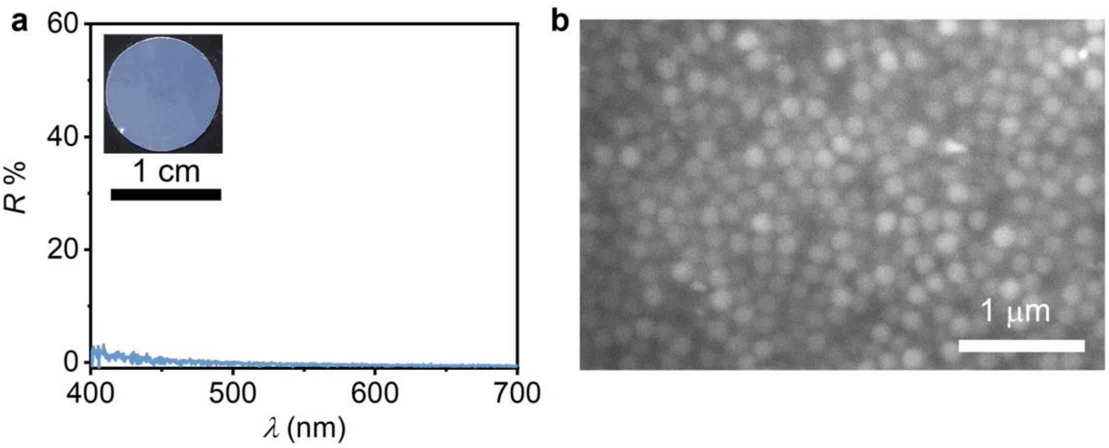

Supplementary Figure 5. The effect of the electrostatic force on ordered structures. a Reflection spectra, digital photo, and **b** SEM image of the MPC fabricated in the presence of ionic liquid (EMBTF, 0.1 M).

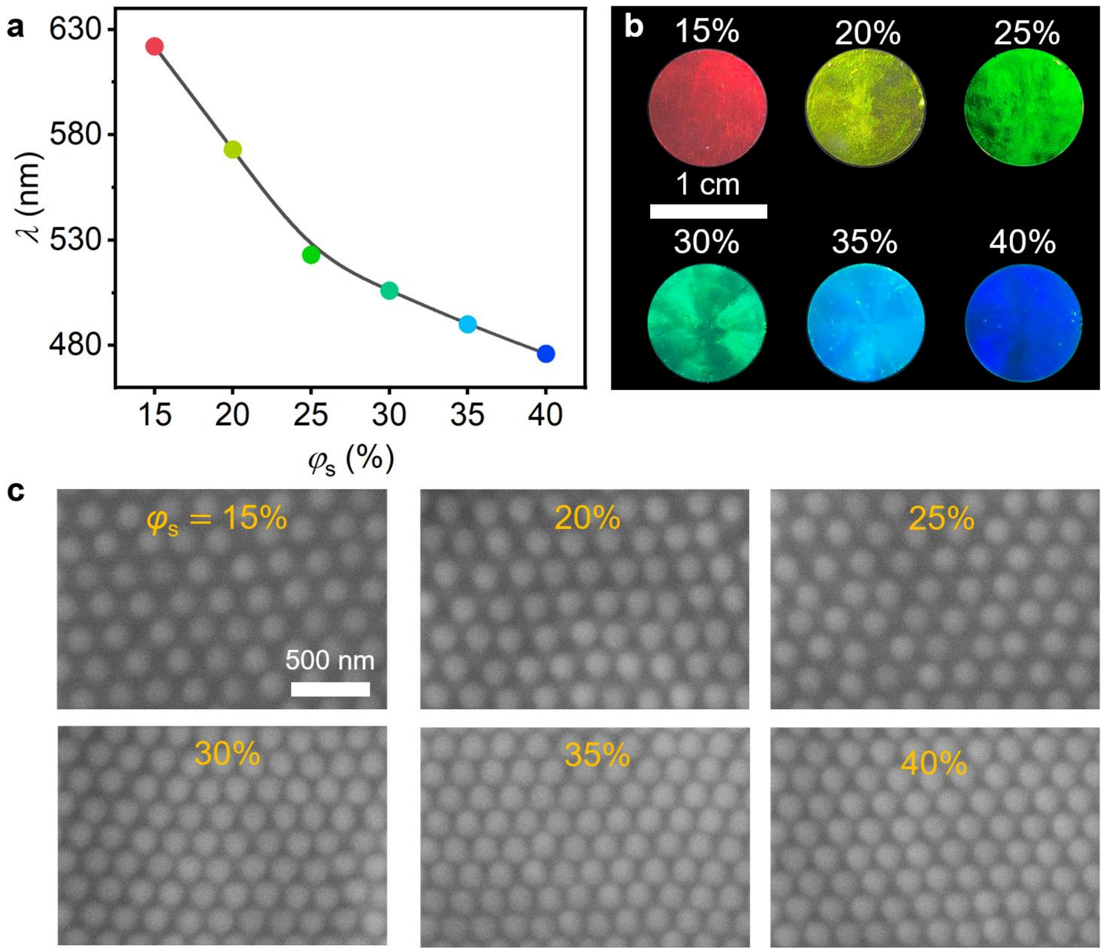

Supplementary Figure 6. The effect of φ_s on the structural color of MPCs. a φ_s as a function of the reflection wavelength. **b** Digital photos and **c** SEM images of MPCs with different φ_s.

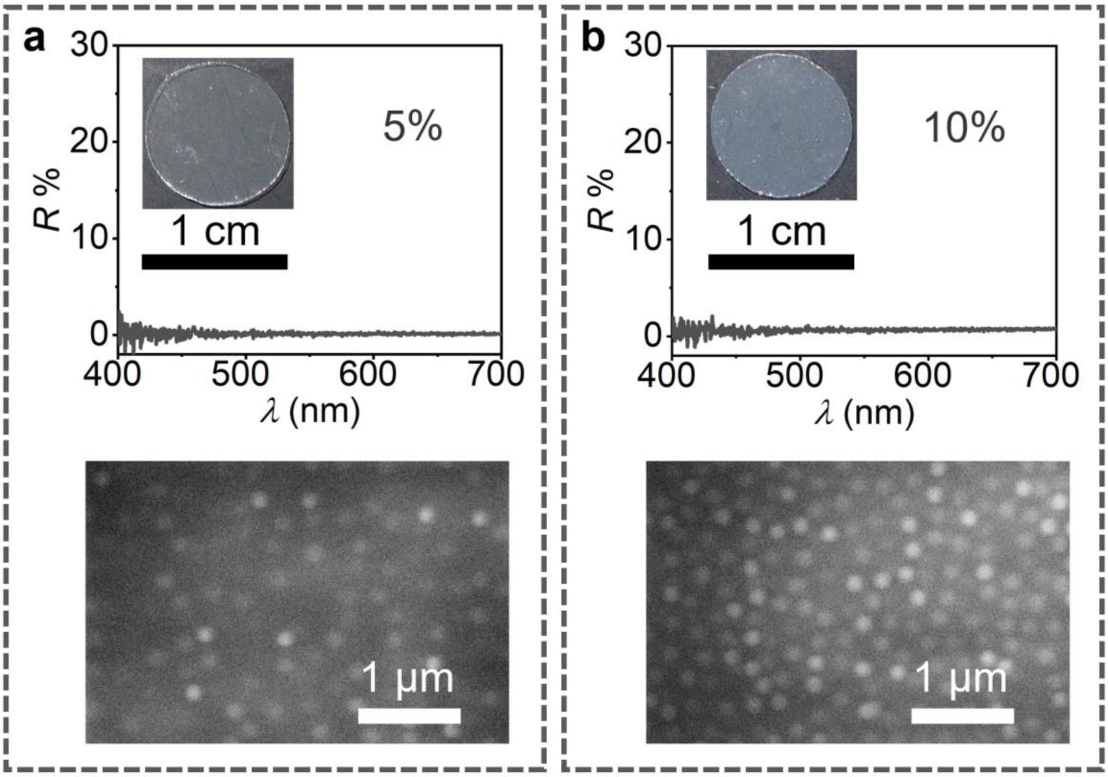

Supplementary Figure 7. Assembly of silica particles when $\varphi_s < \varphi_{th}$. Reflection spectra, digital photos, and SEM images of MPCs with φ_s of **a** 5% and **b** 10%, respectively.

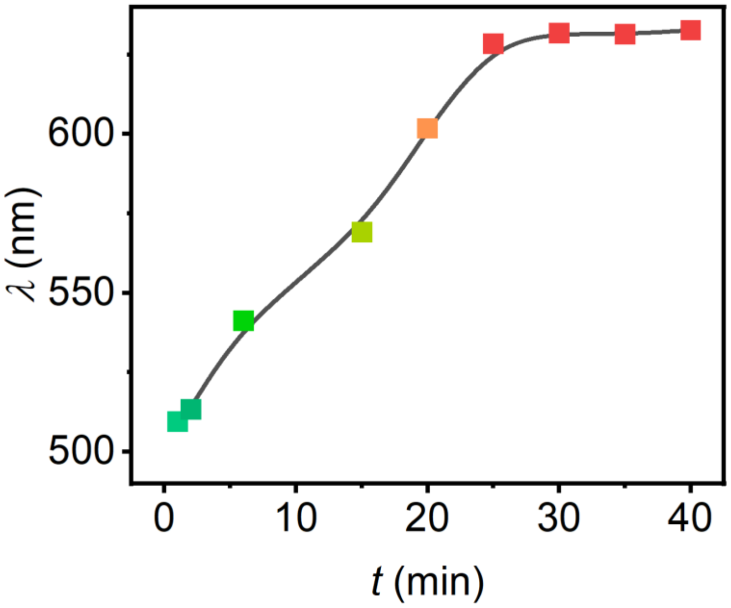

Supplementary Figure 8. Swelling wavelength analysis. The wavelength as a function of the swelling time.

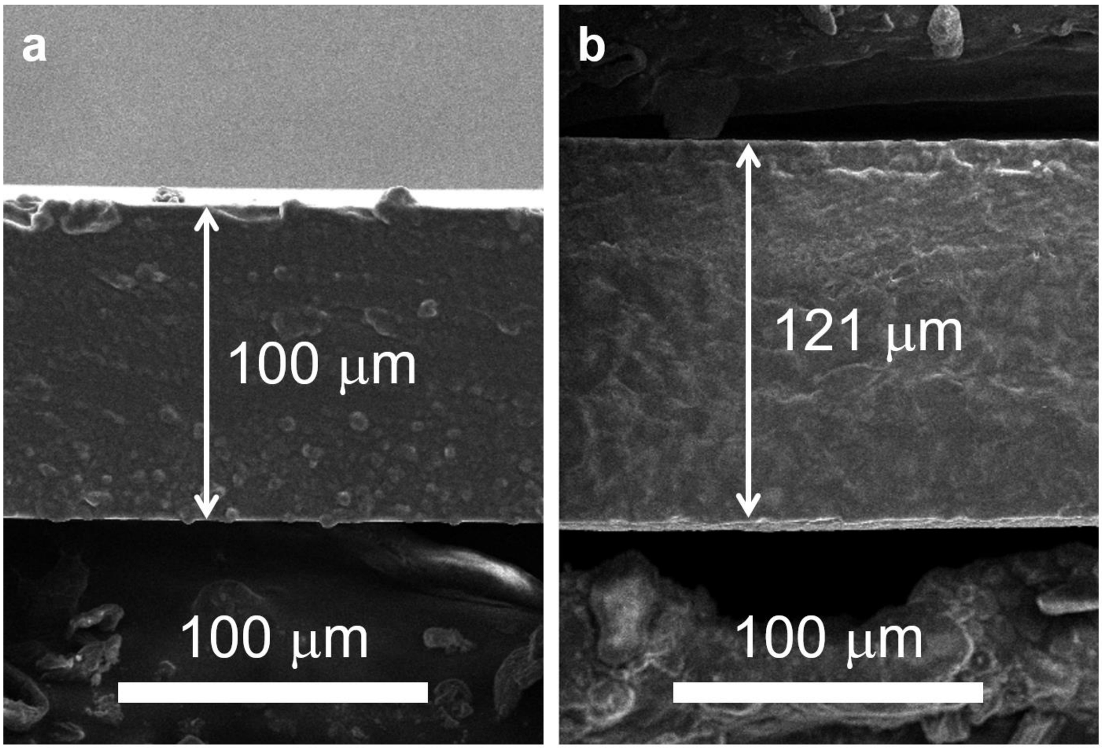

Supplementary Figure 9. Thickness analysis. Cross-sectional SEM images of the **a** MPC and **b** TRMPC.

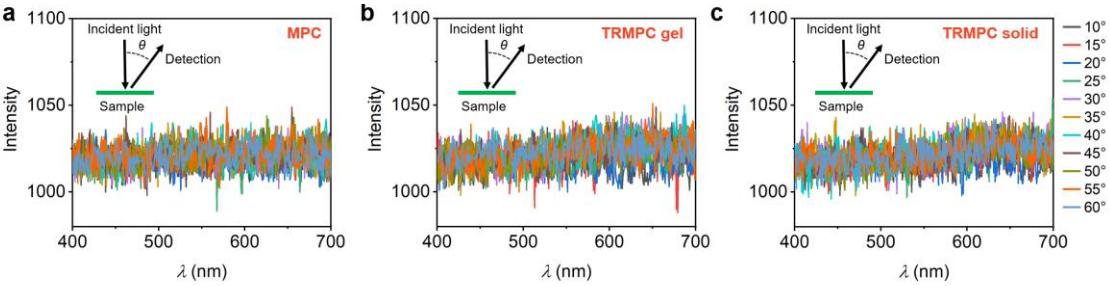

Supplementary Figure 10. Comparison of the scattering between the MPC, TRMPC gel, and TRMPC solid. a-c The scattering spectra of the MPC, TRMPC gel, and TRMPC solid. The incident angle is fixed to 0° and the detection angle varies from 10 to 60° with an interval of 5°.

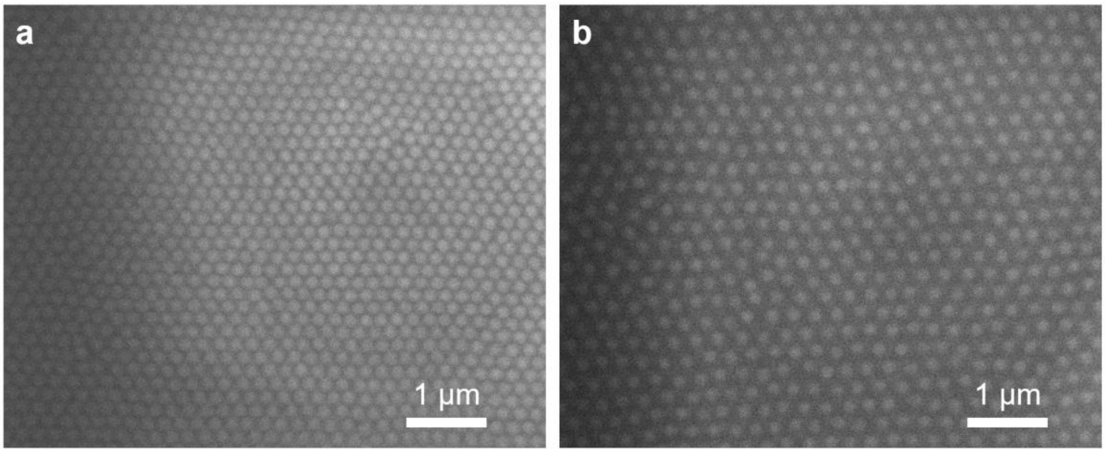

Supplementary Figure 11. Comparison of the order degree between the MPC and TRMPC.

a-b SEM images of MPC and TRMPC, respectively.

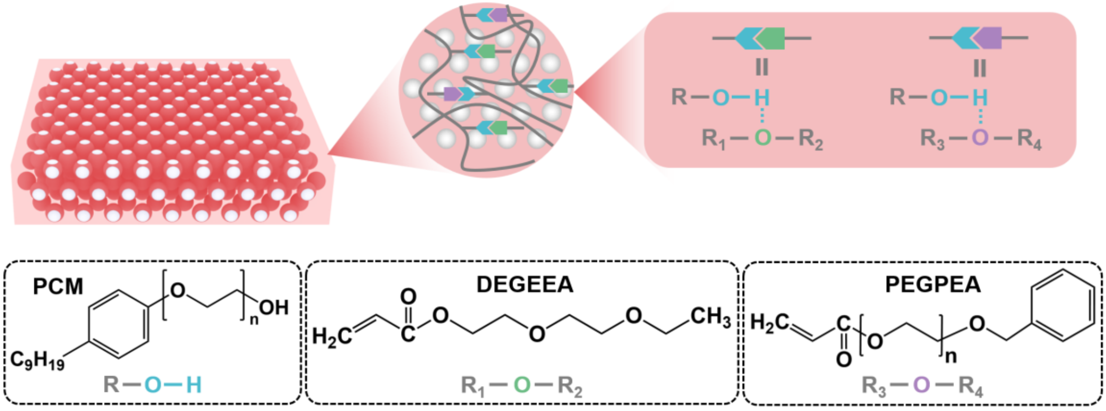

Supplementary Figure 12. The affinity between PCM and MPC. Schematic illustration of the hydrogen bonds between PCM and polymers (PEGPEA and DEGEEA).

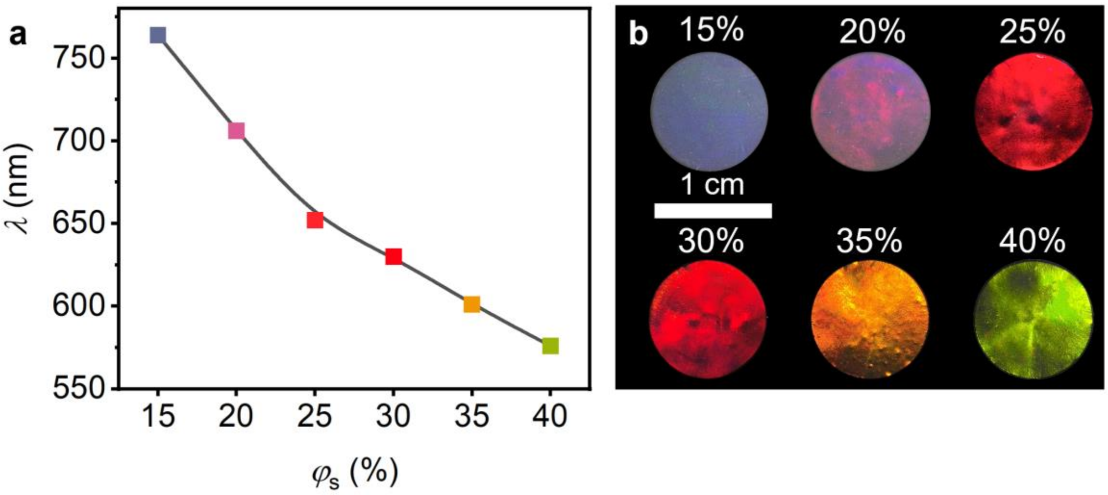

Supplementary Figure 13. The effect of φ_s on structural colors of TRMPCs. a φ_s as a function of the wavelength of the TRMPC gel. **b** The digital photos of the TRMPC gels with different φ_s.

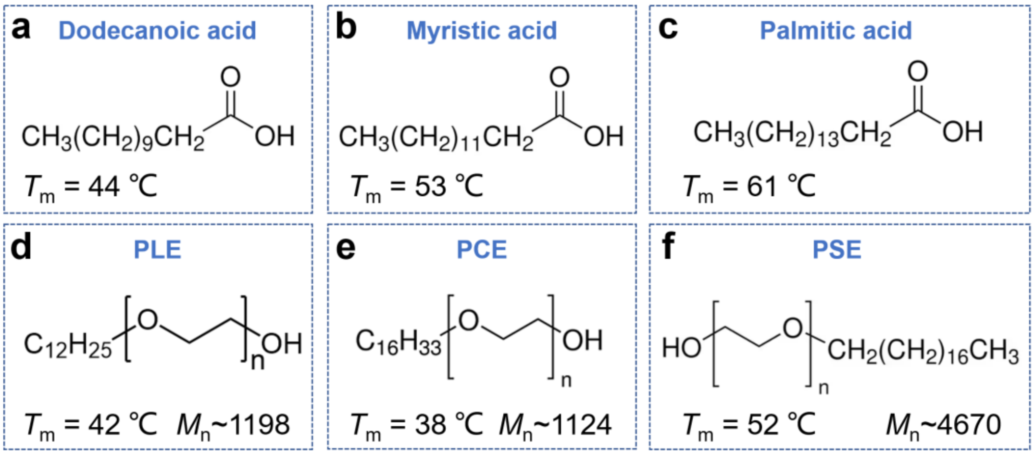

Supplementary Figure 14. Chemical structure of different PCMs. The chemical structure of **a** dodecanoic acid ($CH_3(CH_2)_{10}COOH$), **b** myristic acid ($CH_3(CH_2)_{12}COOH$), **c** palmitic acid ($CH_3(CH_2)_{14}COOH$), **d** polyoxyethylene (23) lauryl ether (PLE, $(C_2H_4O)_nC_{12}H_{26}O$), **e** polyoxyethylene (20) cetyl ether (PCE, $HO(CH_2CH_2O)_{20}C_{16}H_{33}$), and **f** polyoxyethylene (100) stearyl ether (PSE, $C_{18}H_{37}(OCH_2CH_2)_nOH$), respectively.

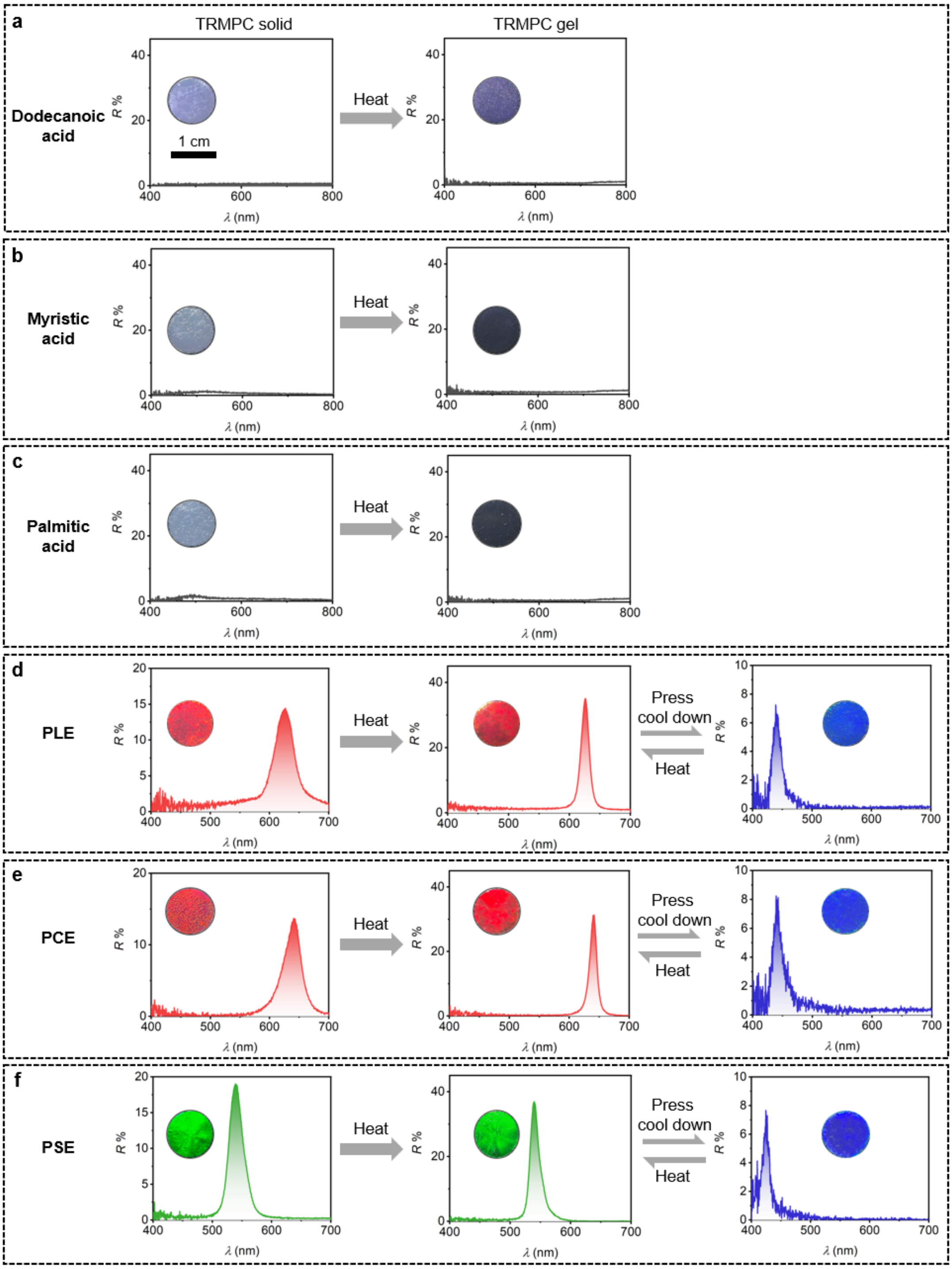

Supplementary Figure 15. TRMPCs with diverse PCMs. The reflection spectra and corresponding digital photos of TRMPCs with different PCMs, **a** dodecanoic acid, **b** myristic acid, and **c** palmitic acid, **d** PLE, **e** PCE, and **f** PSE.

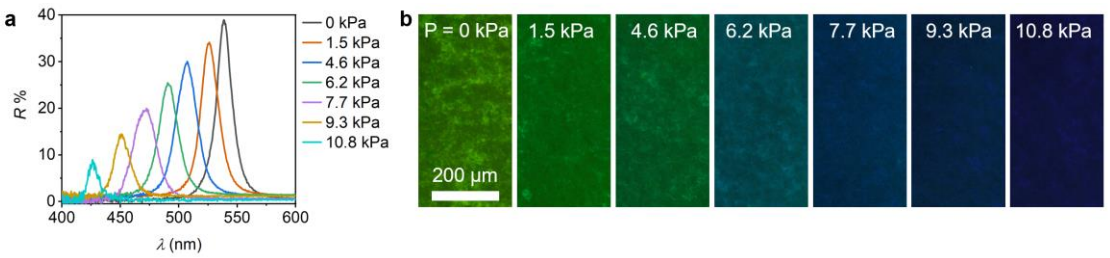

Supplementary Figure 16. Mechanochromic properties of the TRMPC gel prepared by PSE under different pressures. a The reflection spectra and **b** the corresponding microscope images of the TRMPC gel under different pressures.

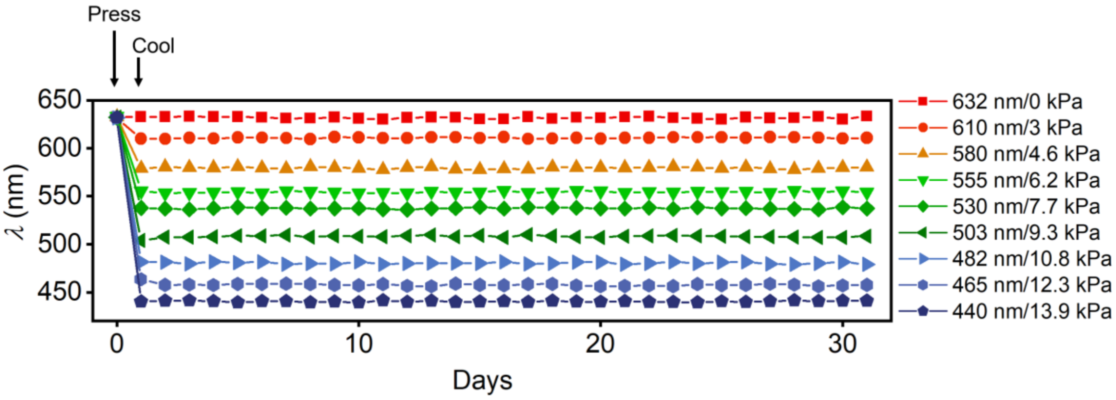

Supplementary Figure 17. Stability of TRMPC solid. The wavelength of TRMPC solid (press to different refection peak position) as a function of time.

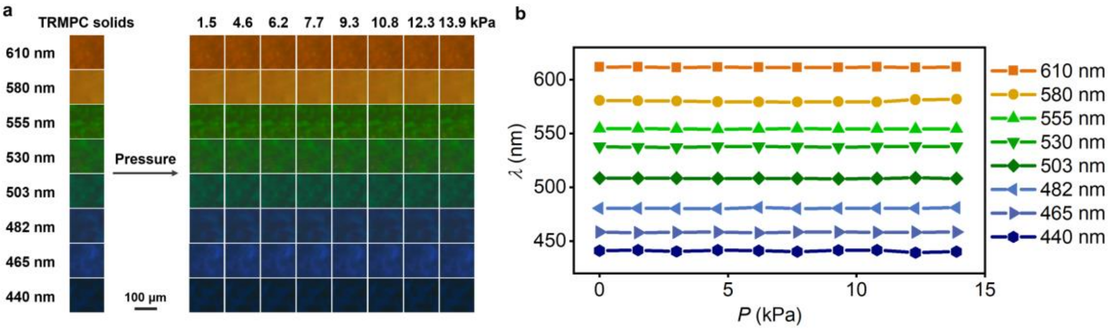

Supplementary Figure 18. The off-state mechanochromism of the TRMPC solid under different pressures. a The microscope images of TRMPCs under different pressures. **b** The λ of TRMPCs solid as a function of pressure.

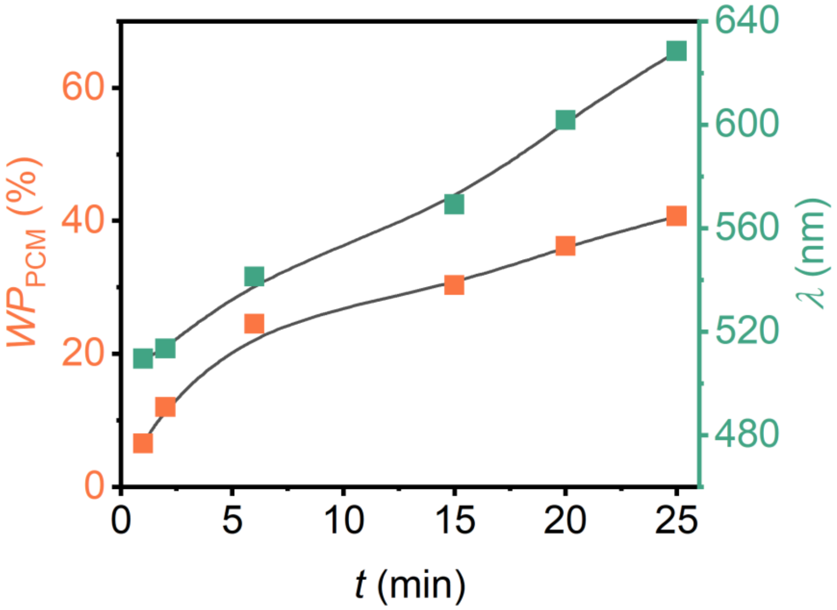

Supplementary Figure 19. The role of the PCM in color recordability. The WP_{PCM} and wavelength of the TRMPC as a function of the swelling time.

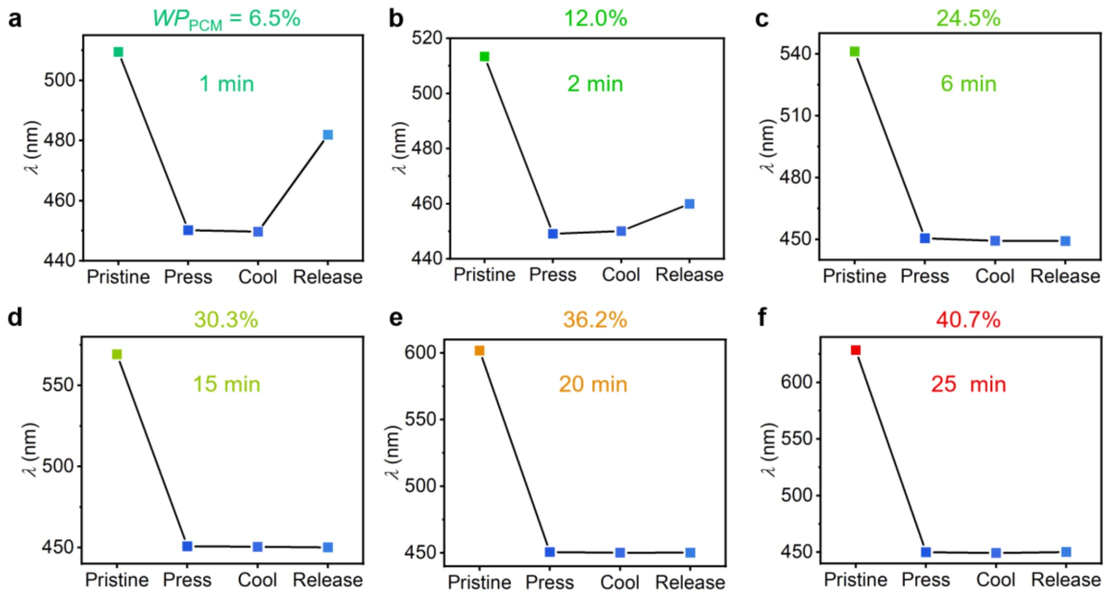

Supplementary Figure 20. Recordability analysis. The wavelength of the TRMPC at different states with the swelling time of **a** 1, **b** 2, **c** 6, **d** 15, **e** 20, and **f** 25 min, respectively.

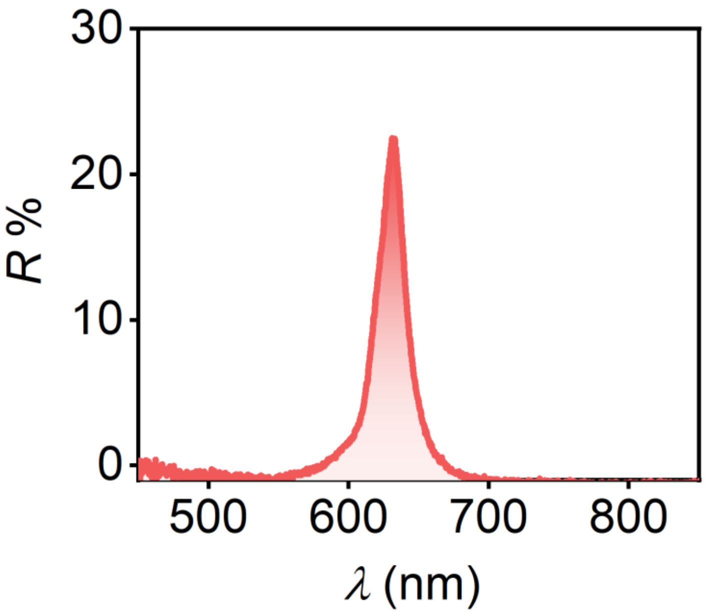

Supplementary Figure 21. Analysis of color recording capability. The reflection spectra of TRMPC solid.

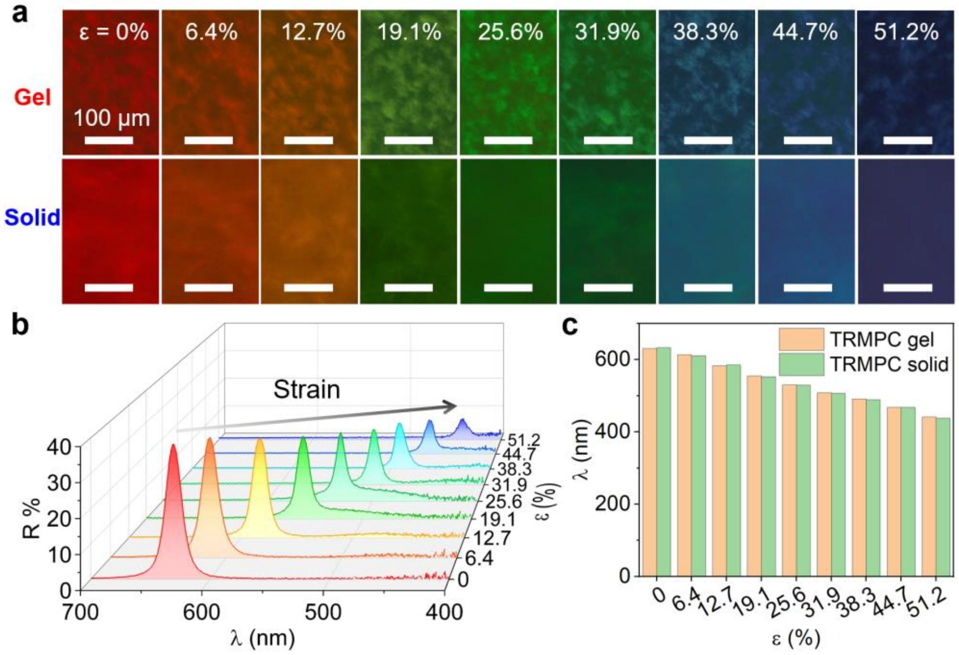

Supplementary Figure 22. Multicolor recordability based on stretching the TRMPC. a The microscope images of TRMPC gel and solid under different strains. **b** The reflection spectra of TRMPC gel under different strain. **c** The λ of TRMPC gel and solid as a function of strain.

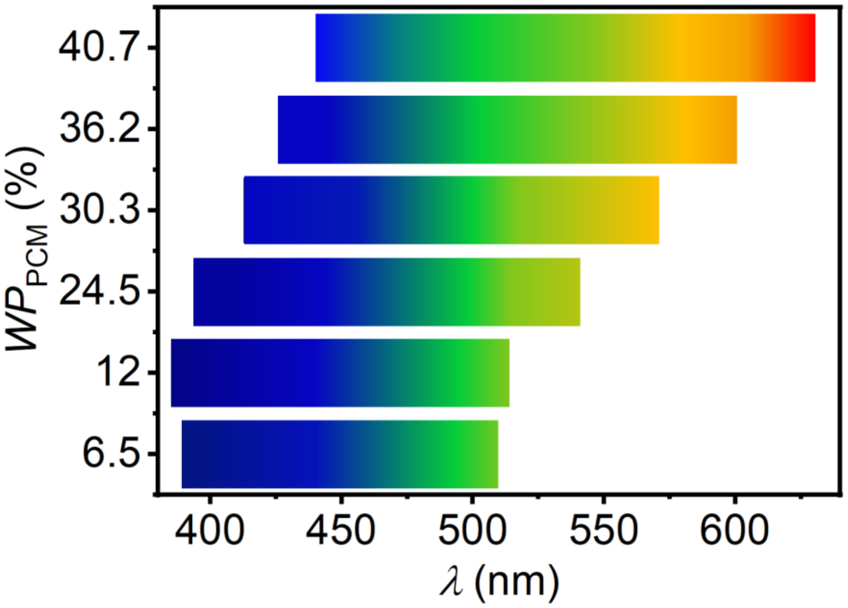

Supplementary Figure 23. Color recording range. The $\Delta\lambda$ of the TRMPC with different WP_{PCM}. The rainbow bars indicate the spectral range within which the reflection peak positions can be varied.

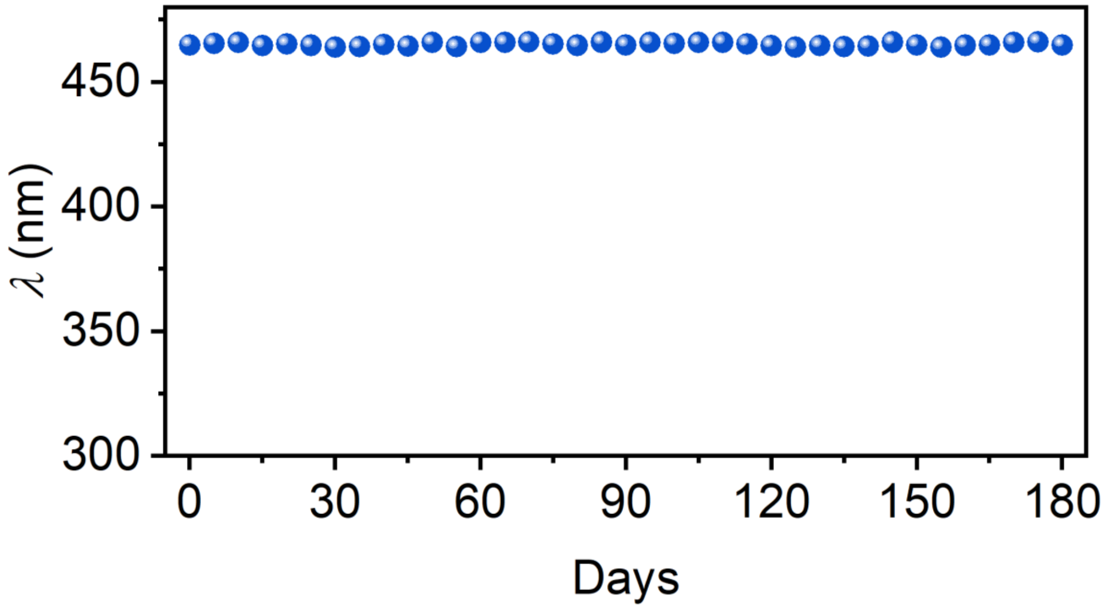

Supplementary Figure 24. Stability of TRMPC. The wavelength of the TRMPC solid over time.

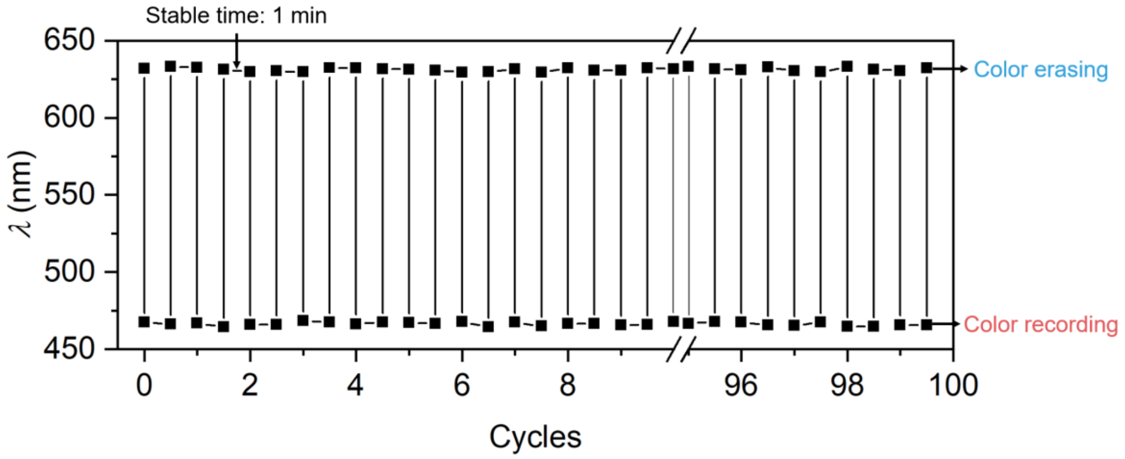

Supplementary Figure 25. Reusability of TRMPC. The number of switching times of the TRMPC solid between 630 and 465 nm.

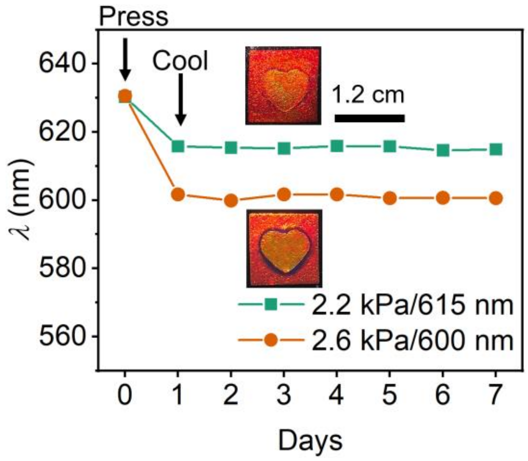

Supplementary Figure 26. The minimal pressure for effective patterns. The λ of patterns as a function of time. The insets are the digital photos of the patterns.

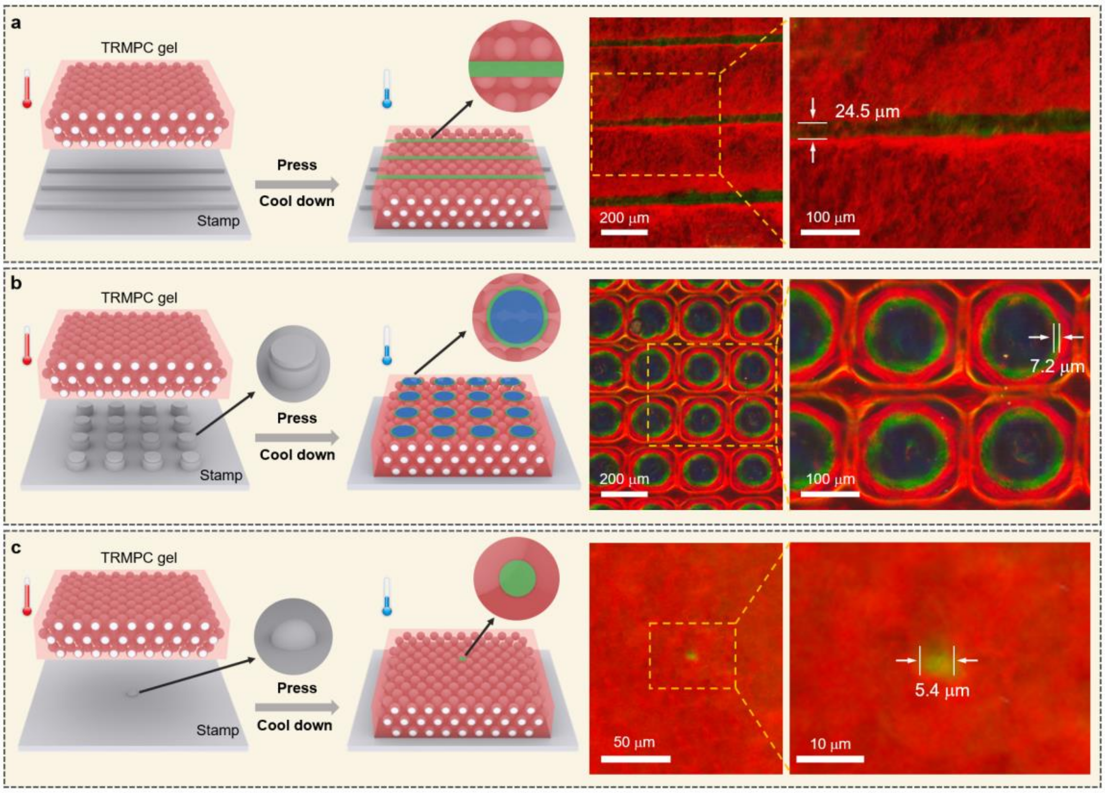

Supplementary Figure 27. The maximum resolution of the patterns. a-c Schematic illustration of the preparation of micropatterns and the corresponding microscope images of micropatterns.

Supplementary Note 1. Calculation of the Δn of the TRMPC and MPC.

The Δn of the TRMPC was calculated by equations 1 and 2. The refractive index (n) of the silica particles (n_s), PEGPEA (n_p), DEGEEA (n_d), and PCM (n_{PCM}) are 1.460, 1.520, 1.470, and 1.505, respectively. For TRMPC, the volume fractions of silica particles (φ_s), PEGPEA (φ_p), DEGEEA (φ_d), and PCM (φ_{PCM}) are 13.2%, 9.3%, 21.6%, and 55.9%, respectively. The Δn of TRMPC is calculated to be 0.038 according to equations 1-2. In comparison, the φ_s, φ_p, and φ_d of the MPC are 30%, 21%, and 49%, respectively, resulting in a Δn = 0.025 calculated by equations 1 and 3. Therefore, the introduction of the PCM with high n into the MPC leads to a slight increase in Δn.

$$\Delta n = n_{\text{silica}} - n_{\text{surroundings}} \quad (1)$$

For TRMPC, $n_{\text{surroundings}} = (n_p\varphi_p + n_d\varphi_d + n_{PCM}\varphi_{PCM})/(\varphi_p + \varphi_d + \varphi_{PCM})$ (2)

For MPC, $n_{\text{surroundings}} = (n_p\varphi_p + n_d\varphi_d)/(\varphi_p + \varphi_d) = n_p f_p + n_d f_d$ (3)

Supplementary Note 2. The effect of Δn (f_p) on reflectivity was investigated without the effect of f_p (Δn).

We first investigate the effect of Δn on reflectivity without changing f_p. The φ_s, φ_p, and φ_d of the MPC are set as 30%, 21%, and 49%, respectively, with f_p = 0.3 and f_d = 0.7. The refractive index (n) of the silica particles (n_s), PEGPEA (n_p), and DEGEEA (n_d) are 1.460, 1.520, and 1.470, respectively. Then, DEGEEA was replaced by other acrylates, including PPGA (n = 1.456, Δn = 0.015), DEGEEA (n = 1.471, Δn = 0.026), PEGDA (n = 1.506, Δn = 0.050), and PETA (n = 1.527, Δn = 0.065) to prepare MPCs with the same f_p (0.3) but different Δn. The SEM images of these MPCs with diverse Δn show similar highly ordered structures (Supplementary Fig. 28a), indicating the change in Δn has a negligible effect on the structural order. The reflectance increases from 15% to 68% when Δn increases from 0.015 to 0.065 (Supplementary Fig. 28b-c), demonstrating that the reflectance is proportional to the Δn. Besides, the λ also increases slightly as Δn increases, which can be attributed to the slight increase in the effective n of MPCs.

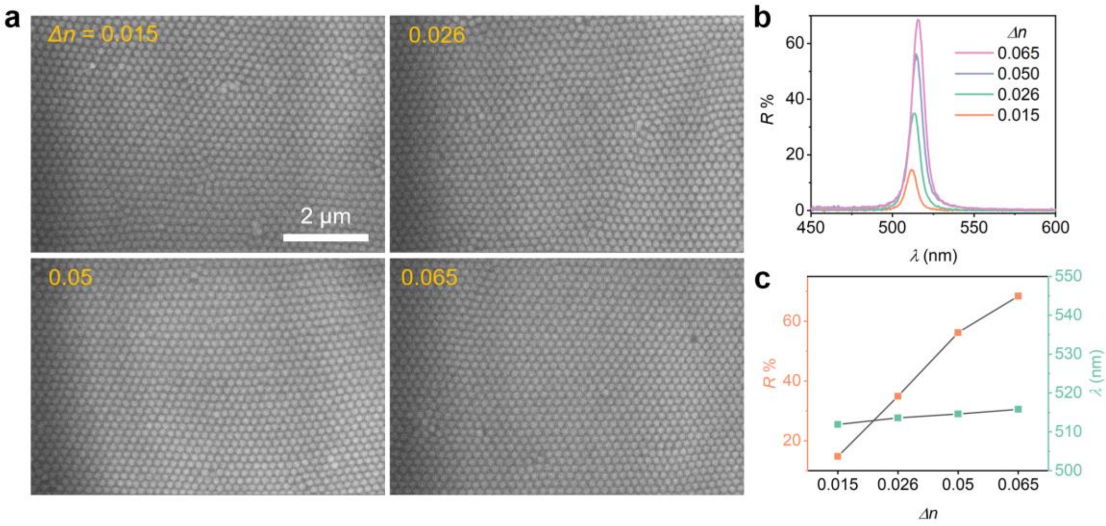

Supplementary Figure 28. The effect of *Δn* on the MPC's reflectance. a SEM images of MPCs with different *Δn*. **b** Reflection spectra of MPCs with different *Δn*. **c** The reflectance and *λ* of MPCs as a function of *Δn*.

We then investigate the effect of f_p on the reflectance without altering *Δn*. For the silica/PEGPEA/DEGEEA MPC, it is impossible to change the f_p only without influencing its *Δn*. In principle, we can fabricate the silica/PEGPEA MPC, and thus investigate the influence of f_p without changing *Δn*, because the silica particles are surrounded by only PEGPEA ($\varphi_p = f_p$). For the silica/PEGPEA MPC, its *Δn* is always a constant value (0.06) regardless of φ_p. As shown in Supplementary Fig. 29, when φ_p = 0.78 and 0.80, the corresponding MPCs exhibit an amorphous structure, leading to negligible reflectance and structural color. The amorphous structures originate from the weak electrostatic repulsion between silica particles caused by the large interparticle distance. When φ_p gradually decreases to 0.72, the interparticle distance decreases, leading to the increase in electrostatic repulsion, order degree, and hence reflectance. As φ_p = 0.70, the reflectance is similar to that of φ_p = 0.72 owing to their similar order degree. Therefore, the reflectance of the silica/PEGPEA MPC is also proportional to the φ_p-dependent order degree. The decrease in φ_p will cause the increase in interparticle distance and thus the decrease in *λ* according to Bragg's law.

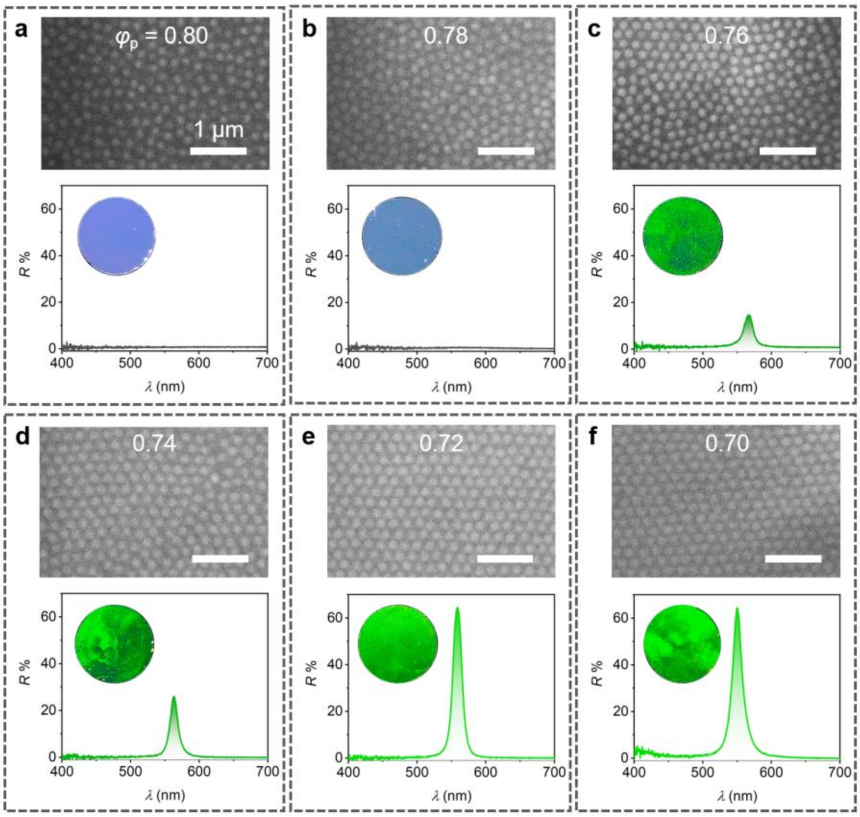

Supplementary Figure 29. Effect of φ_P on the reflectance of MPCs. a-f SEM images, digital photos, and reflection spectra of MPCs with different φ_P. The diameter of all the samples is 1 cm.

Supplementary Note 3. Comparison of materials, structures, and properties between references and this work.

Reference 1 reported a shape memory retroreflection structural color film (SMRSCF) with a micrometer-sized (5-20 μm) dome monolayer array structures and showed its applications in generating patterns with the on-off color contrast. The SMRSCF exhibits interesting retroreflective structural color based on the combined effect of total internal reflection (TIR) and interference. Nevertheless, this specific color generation mechanism leads to unsatisfied optical performances: 1) more than one reflection peaks and thus poor color purity, and 2) no obvious regularity between the color and the size of the dome. The SMRSCF exhibits colorless recordability-erasability based on the SMRSCF's shape memory effect. The colorless recordability involves color disappearance along with the collapse of dome structures by pressing the SMRSCF above the glass transition temperature (T_g: 63 °C) and fixing the colorless state by cooling down. Then, the recorded colorless

state can return to the pristine state after being heated at 120 °C for 10 min. Reference 1 exhibits significant novelty within the areas of focus on the on-off retroreflective colors based on shape memory micro-domes. However, challenges, including impure colors, the difficulty in printing multicolor patterns, the requirements of high pressure (≈ 4 MPa), and the long time for colorless recording (35 min)-erasing (10 min) may limit SMRSCFs' practical applications.

Reference 2 developed core-shell polymer particles-based shape memory photonic crystals (SMPCs) with close-packing structures as smart labels for environmental monitoring, which were fabricated by a facile hot-pressing method and post-photocuring technology. These SMPCs show mechanochromic and thermochromic properties owing to the soft shape memory shell of the polymer particle. Color recordability was realized by stretching the SMPC above the T_g (~ 53 °C) to adjust colors and subsequently fixing changed colors by cooling the stretched SMPC below the T_g. The fixed color could afterward be recovered by heating the stretched SMPC above the T_g for ~ 50 s. This work provides an interesting idea for preparing SMPCs by adjusting the properties of core-shell particles. Nevertheless, the SMPC suffers from a small tuning range of λ ($\Delta\lambda$ = 120 nm), limited sensitivity (2.4 nm/%), low recovery time (~ 50 s), and a dramatic decrease in color saturation during stretching, restricting their practical applications as multicolor rewritable papers.

Reference 3 reported a stimuli-responsive shape-memory polymer (SMP) with inverse opal structures, which can realize cold programming and instantaneous shape recovery at the nanoscale. The pristine SMP exhibits brilliant color due to the ordered packing of micropores and large refractive index contrast between air and polymers. The macropores of the SMP collapse once compressing the SMP, leading to the disappearance of structural color and a transparent appearance. This specific working mechanism means that patterns with only a monochrome color can be recorded on the SMP paper. The recorded color can revert to the pristine state by immersing compressed SMPs in solvents with low surface tension (ethanol and toluene). Unfortunately, the monochrome color recordability and the use of organic vapors may limit their large-area applications.

Reference 4 proved the fabrication of a rewritable PC paper based on programmable configurations of a synthesized shape memory polymer (SMP). The SMP-based PC paper possesses compression-induced collapsed micropores, thus showing a colorless state. The collapsed micropores can be partially or fully recovered by controlling the time of laser-induced heating,

which results in different colors. Multicolor patterns can be generated and recorded by region-selectively heating the PC papers to different temperatures using lasers and integrating a pre-printed photothermal layer. The recorded color can be erased by simply compressing the whole PC paper. This work offers a possibility to record different colors by controlling the recovery degree of the PC's micropores. However, the unwanted diffusion colors caused by the inevitable conduction of heat at boundaries will restrict the color purity and the resolution of patterns.

Reference 5 reported elastoplastic inverse opals with the polymer SU-8 fabricated by infiltrating the colloidal crystals of silica particles with uncrosslinked SU-8 followed by the removal of the colloidal templates. The SU-8 films show a mechanochromic response that can change and record the reflective color depending on the magnitude and rate of the applied force. Due to the elastoplastic deformation of the SU-8 films, the deformed structures and thus colors can be locked after removing the load (17.6-20.4 MPa). Nevertheless, massive cracks on the SU-8 films lead to poor color purity. Moreover, the recorded colors cannot be erased due to the permanent structure change, which is unfavorable for rewritable materials.

Therefore, references 1-4 majorly reported the SMP-based photonic materials with closely arranged micro-dome monolayer, closely packed opal structures, and inverse opal structures. The keys to realizing color recordability and erasability are based on the intrinsic shape memory effect. In contrast, reference 5 reported the specific SU-8 with inverse opal structures capable of recording colors but not available for erasing colors, owing to the irreversible elastoplastic deformation of the SU-8 films.

In contrast, our work developed a type of thermal-responsive mechanochromic photonic crystal (TRMPC) by combining phase-change materials (PCM) with a high melting point (T_m: 46 °C) and mechanochormic photonic crystals (MPCs) with non-closely packing structures and demonstrated its applications as ink-free rewritable paper for printing multicolor patterns. Owing to the efficient light diffraction by the non-close-packing structure with a small refractive index contrast, the TRMPC exhibits outstanding color purity and a broad tuning range of structural colors, favorable for practical applications. More importantly, the TRMPC shows multicolor recordability-erasability based on the thermoswitchable on-off mechnanochromism. The color recordability is accomplished in two processes: color generation by pressing the TRMPC at $T > T_m$ and subsequently color fixation through a cooling down process ($T < T_m$). Multicolor can be recorded by adjusting

pressure with similar procedures. The recorded color can be erased by heating the TRMPC at 80 °C in seconds. High-resolution patterns (line: 7.2 μm and dot: 5.4 μm) can be repeatedly printed on the TRMPC paper.

The above discussion proves the significant differences between our work and references 1-5 in material fabrication, structure design, color-generating mechanism, optical properties, color recordability-erasability, and printing patterns (Table 1).

Supplementary Table 1. Comparisons of materials, structures, and properties between references 1-5 and our work.

	Ref 1	Ref 2	Ref 3	Ref 4	Ref 5	Our work
Materials	Epoxy resin/PU/PA	PS@PEA@PiBMA-co-PEA	ETPTA-co-PEGDA	PS-co-BA	SU-8	SiO_2/PEGPEA/DEGEEA/PCM
Structure	Closely arranged micro-dome monolayer	Close-packing opals	Inverse opal	Inverse opal	Inverse opal	Non-close-packing opals
Color-generating mechanism	TIR and interference	LD by opals	LD by inverse opals	LD by inverse opals	LD by inverse opals	LD by opals
Structural color	Retroreflection	Reflection	Reflection	Reflection	Reflection	Reflection
Color purity	Poor	Good	Good	Poor	Poor	Good
Working mechanism	Shape memory effect	Shape memory effect	Shape memory effect	Shape memory effect	Elastoplastic deformation	Thermoswitchable on-off mechanochromism
Tuning range of λ	×	120 nm	×	143 nm	165 nm	192 nm
Multicolor	×	N.A	×	√	√	√
Patterning requirements	~4 MPa<br>$T > T_g$<br>> 35 min	5-50 %<br>$T > T_g$<br>N.A	4.21-54.4 kPa<br>23 °C<br>N.A	~18 MPa<br>$T > T_g$<br>2-10 s	17.6-20.4 MPa<br>25 °C<br>0.3 s	3-12.3 kPa<br>$T > T_m$<br>< 1 min

Erasing	120 ℃	53 ℃	23 ℃	80 ℃	25 ℃	80 ℃
requirements	10 min	~ 50 s	N.A	8 min	×	1.8 s
Selective erasure	N.A	N.A	N.A	N.A	×	√
Resolution	N.A	N.A	N.A	N.A	N.A	7.2 μm
Rewriteability	√	N.A	√	√	×	√

*N. A. is not available. TIR and LD represent total internal reflection and light diffraction, respectively.

Supplementary References

1. Ji, C., Chen, M. & Wu, L. Patternable and rewritable retroreflective structural color shape memory polymers. *Adv Optical Mater* **9**, 2100739 (2021).
2. Wu, P., Shen, X., Schafer, C. G., Pan, J., Guo, J. & Wang, C. Mechanochromic and thermochromic shape memory photonic crystal films based on core/shell nanoparticles for smart monitoring. *Nanoscale* **11**, 20015-20023 (2019).
3. Fang, Y., Ni, Y., Leo, S. Y., Taylor, C., Basile, V. & Jiang, P. Reconfigurable photonic crystals enabled by pressure-responsive shape-memory polymers. *Nat. Commun.* **6**, 7416 (2015).
4. Wang, Y., Zhao, Q. & Du, X. Inkless multi-color writing and copying of laser-programmable photonic crystals. *Mater. Horiz.* **7**, 1341-1347 (2020).
5. Cho, Y. et al. Elastoplastic inverse opals as power-free mechanochromic sensors for force recording. *Adv. Funct. Mater.* **25**, 6041-6049 (2015).
