# Supplementary material for: Multicolor recordable and erasable photonic crystals based on on-off thermoswitchable mechanochromism toward inkless rewritable paper
Source: Nat Commun. 2024 Jul 5;15:5643. doi: 10.1038/s41467-024-49860-3 (PMC11226673; doi:10.1038/s41467-024-49860-3)
Supplement: Supplementary file 3 — Description of Additional Supplementary Files [file 41467_2024_49860_MOESM3_ESM.pdf]

Description of Additional Supplementary Files

File Name: Supplementary Movie 1
Description: The printing process of PC pattern.

File Name: Supplementary Movie 2
Description: The erasing process of PC pattern.
